# Expression patterns of the immune checkpoint ligand CD276 in urothelial carcinoma

**DOI:** 10.1186/s12894-021-00829-0

**Published:** 2021-04-12

**Authors:** Wilhelm K. Aicher, M. Korn, L. Reitnauer, F. B. Maurer, J. Hennenlotter, P. C. Black, T. Todenhofer, J. Bedke, A. Stenzl

**Affiliations:** 1 Department of Urology, University of Tuebingen Hospital, Hoppe-Seyler-Str. 3, 72076 Tübingen, Germany; 2 Vancouver Prostate Centre, University of British Columbia, Level 6, 2775 Laurel St, Vancouver, BC V5Z 1M9 Canada; 3 Department of Urology, Center for Medical Research, University of Tuebingen Hospital, Waldhoernlestrasse 22, 72072 Tübingen, Germany

**Keywords:** Urothelial carcinoma, Immune check-point protein, CD276, B7-H3, Bladder tumor stages

## Abstract

**Background:**

CD276 is an immune checkpoint molecule. Elevated CD276 expression by urothelial carcinoma is associated with poor prognosis, but little is known about its expression across different tumor stages. We therefore investigated CD276 expression in bladder cancer (BC) cells and in tissue samples of BC stages from pT2 to pT4.

**Methods:**

CD276 expression was explored in 4 urothelial cancer cell lines and 4 primary normal urothelial cell populations by quantitative RT-PCR, Western blot and flow cytometry. CD276 was investigated in bladder tumors from 98 patients by immunohistochemistry using a score (0–300) incorporating both, staining intensity and area of CD276 staining. Normal appearing urothelium in the bladder of the same patients served as controls.

**Results:**

The urothelial carcinoma cell lines expressed significantly higher levels of CD276 on transcript (*p* < 0.006), total protein levels (*p* < 0.005), and on the cell surface (*p* < 0.02) when compared to normal urothelial cells. In pT2–T4 tumor tissue samples, CD276 was overexpressed (median score 185) when compared to corresponding healthy tissues from the same patients (median score 50; *p* < 0.001). No significant differences in CD276 expression were recorded in late, locally advanced ≥ pT3a tumors (median score 185) versus organ-confined < pT3a tumors (median score 190), but it was significantly lower in the normal urothelial tissue associated with ≥ pT3a tumors (median score 40) versus < pT3a tumors (median score 80; *p* < 0.05).

**Conclusion:**

CD276 expression is significantly elevated in urothelial carcinoma cells in all stages but varies between individuals considerably. Reduced CD276 expression in normal urothelial cells may imply that these cells would be protected from CD276-mediated immuno therapies.

## Background

Bladder cancer ranks among the most common malignancies. Despite efforts in cancer research, the 5-year survival rates between 1996–2004 (80%) compared to 2008–2014 (77%) have not improved [1]. Therefore, additional tools for diagnosis and more effective therapies are urgently needed.

Recently active components to modulate immune responses have emerged as promising cancer therapies in bladder carcinoma [2]. These therapies mainly target the programmed death receptor 1 (PD-1) and its respective ligand PD-L1. PD1/PD-L1 immune checkpoint inhibitors have been approved in clinical settings for treatment of patients who are either cisplatin-refractory or cisplatin-ineligible based on the results of large phase II–III trials [3–5]. Other immune checkpoint receptors have been described and represent additional promising targets for therapy.

One such attractive therapeutic target is CD276 (alias B7-H3) which is over-expressed in many tumors [6–8] and often correlates with poor prognosis [9–11]. CD276 is a member of the B7/CD28 family of antigen independent co-stimulatory ligands. In mice, CD276 binds to the triggering receptor expressed on myeloid cells like transcript 2 (TLT-2) protein to modulate T-cell responses [12]. Elevated expression of CD276 enhances the TLT-2-mediated CD8^+^ cytotoxic T-lymphocyte response to tumor in mice [13]. In another study, CD276 prevented activation of anti-tumor responses by blocking CD4^+^Th_1_- activation [14]. There is experimental evidence promising success of anti-CD276 tumor therapies [6, 14–16], and several clinical studies are underway or already completed (see https://clinicaltrials.gov).

Targeting CD276 directly on tumor cells is a promising strategy [6]. Such targeting would require knowledge about the amount of receptor on the cells and its distribution in the tissue. It has been estimated that 2000 CD276 ligands would be required per tumor cell for such a targeted therapy to be effective [6]. In this context, the objective of this study was to study the expression levels of CD276 on well-characterized human urothelial cancer cell lines and in tissue samples from bladder tumors as a function of the tumor staging.

## Methods

### Preparation and expansion of cells

Urothelial cancer cell lines (UCCLs) HT1197 (CRL-1473), TCCsup (HTB-5), RT4 (HTB-2), and 5637 (HTB-9) were purchased from ATCC (www.atcc.org). They were expanded in MEM medium enriched with bovine serum, non-essential amino acids, sodium pyruvate and antibiotics as recommended by the supplier. Normal urothelial cells (NUCs) were prepared from cancer-free ureteral tissue samples from 4 donors after informed and written consent, and expanded as previously described [17]. The integrity of each NUC culture was confirmed by immunohistochemistry with the pancytokeratin marker cytokeratin AE1/AE3 (not shown). Contamination of cultures by mycoplasma were excluded by PCR analysis following established protocols (Biontex). The study was approved by the Ethics Committee under file number 341/2002/BO.

### Analysis of CD276 transcript and protein expression

Cells were expanded to reach confluence, washed with cold PBS, detached by mild proteolysis, lysed to isolate RNA (Qiagen RNeasy kit), and cDNA was synthesized by reverse transcription and oligo(dT) priming (Takara Bio). Quantitative RT-PCR was performed employing CD276 target gene primers. GAPDH and PPIAγ amplifications served as internal controls (Table 1) [18]. Proteins were detected in cell lysates after SDS-PAGE by immunoblotting (IB) [19] using monoclonal antibodies (mAb) or sera, followed by peroxidase-labelled secondary antibodies (Table 2), and quantified by a blot scanner (C-Digit, Licor). The CD276 signal intensities in each sample were normalized to β-actin signals of each sample.

**Table 1:** Primers for quantitative RT-PCR

Gene	Primer	Sequence (5′ → 3′)
CD276	Forward	TTTCCTTTCCCCTCC
	Reverse	TGTGACCAGCACATG
GAPDH	Forward	GAGTCAACGGATTTGGTCGT
	Reverse	TTGATTTTGGAGGGATCTCG
PPIAγ	Forward	TTCATCTGCACTGCCAAGAC
	Reverse	TCGAGTTGTCCACAGTCAGC

**Table 2:** Antibodies to human antigens and detection reagents for immunoblotting (IB), flow cytometry (FC), and immunohistochemistry (IHC)

Target	Antibody/label	Species	Clone	Dilution	Source	Order #
*IB*						
CD276	IgG1 mAB	Mouse	6A1	1:600	abcam	ab105922
CD276	serum	Rabbit	ø	1:500	abcam	ab226256
β actin	serum	Rabbit	ø	1:1000	abcam	ab8227
Mouse IgG	serum/HRP	Rabbit	ø	1:2000	Dako	P0260
Rabbit Ig	serum/HRP	Goat	ø	1:2000	Dako	20034870
*FC*						
CD276	IgG1 mAB/PE	Mouse	MIH42	1:20	BioLegend	B7RP2
KLH	IgG1 mAB/PE	Mouse	X40	1:20	BD	345816
*IHC*						
CD276	IgG1 mAB	Mouse	6A1	1:300	abcam	ab105922

### Analysis of CD276 expression on cell surfaces

To quantify CD276 expression on UCCLs and NUCs, cells were grown to confluence, detached by mild proteolysis and washed twice by PBS. 5E05 cells were resuspended in PFEA buffer, incubated with anti-CD276 mAB and measured by flow cytometry (FC) as described (LSR II, Becton Dickinson) [20]. Staining of cells with a mAB to KLH served as isotype control (Table 2). Signal intensities were normalized to COMP-beads (Becton Dickinson) and the KLH isotype controls in each experiment.

### Detection of CD276 in paraffin sections of tissue microarrays

Tissue microarrays (TMA) were assembled from 0.6 mm bladder tissue punches after radical cystectomy of 98 BC patients after informed and written consent as described [21]. 98 tissue punches were obtained from pathologically confirmed tumor areas. In addition, from the same bladders, 47 tissue punches were prepared from tumor adjacent but healthy appearing areas, which were pathologically confirmed as normal tissue. For immunohistochemistry (IHC), TMA sections were incubated with anti-CD276 mAb (1 h, 20 °C; Table 2), washed three times with 0.1% Tween 120 in PBS and counterstained by OptiView DAB IHC detection kit as described by the supplier (Ventana, Roche, Switzerland). To confirm histology, samples were stained after IHC by hematoxylin and eosin (HE). Antibody staining intensities (no = 0, weak = 1, middle = 2, strong = 3) and the percent area of staining per TMA (0–100%) were recorded by microscopy to generate scores ranging from “0” (no antibody stain) to “300” (maximal staining intensity in the whole TMA) [21].

### Statistics

Experimental results were processed by spreadsheet (Excel®, Microsoft) and statistics software (JMP®, SAS Institute Inc., Cary, USA). P values less than 0.05 were considered significant and marked in the figures accordingly.

## Results

### Expression of CD276 in urothelial carcinoma cell lines in vitro

**Fig. 1:**
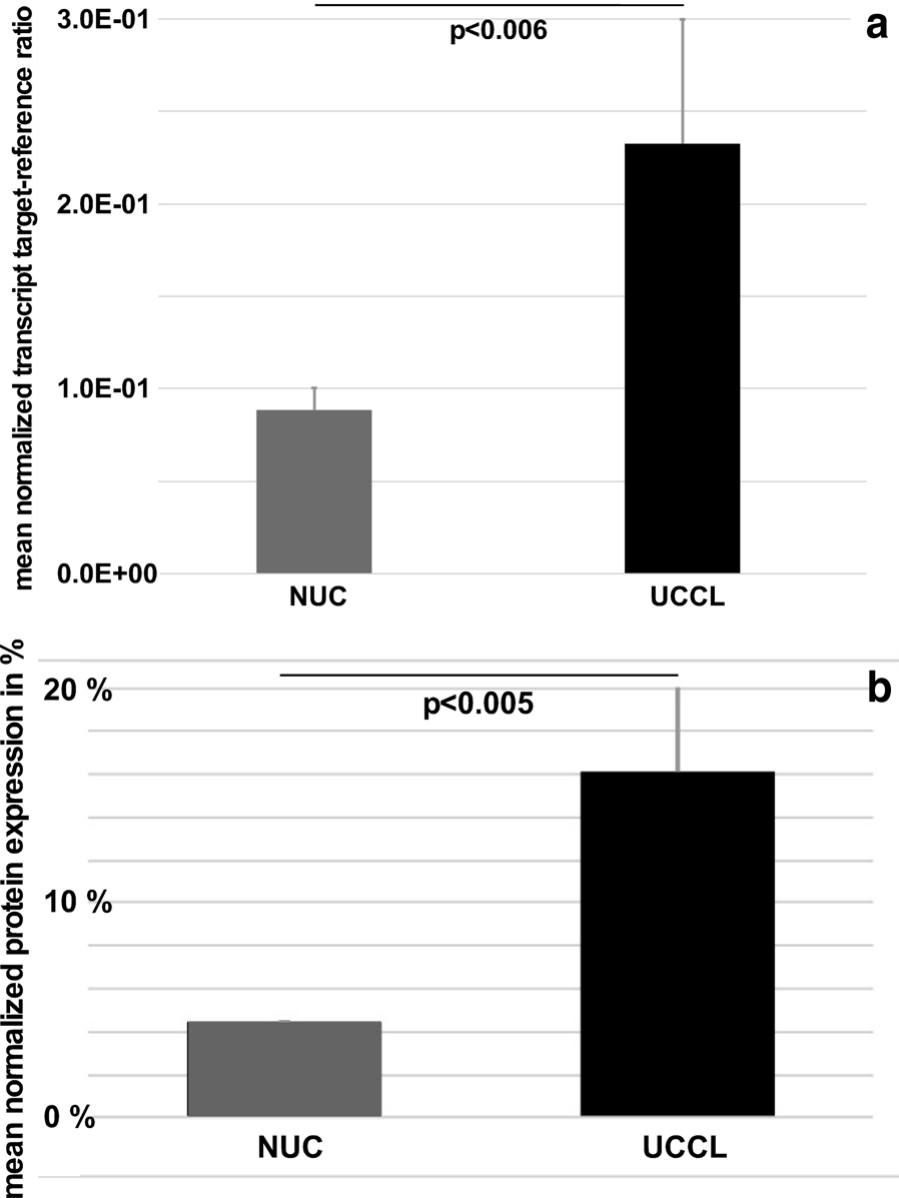
CD276 transcript and protein expression. **a** Steady state mRNA amounts encoding CD276 were enumerated by qRT-PCR in four normal urothelial cells (NUCs) and four urothelial cancer cell lines (UCCLs). The graph shows the mean transcript levels ± standard deviations normalized to two housekeeping genes. **b** The total protein expression was investigated by immuno blotting of SDS-PAGEs. The mean expression of CD276 ± standard deviation of the four NUCs and four UCCLs is normalized to β-actin expression in each individual sample. UCCLs express significantly more CD276 transcripts (*p* < 0.006) and protein (*p* < 0.005) when compared to NUCs

**Fig. 2:**
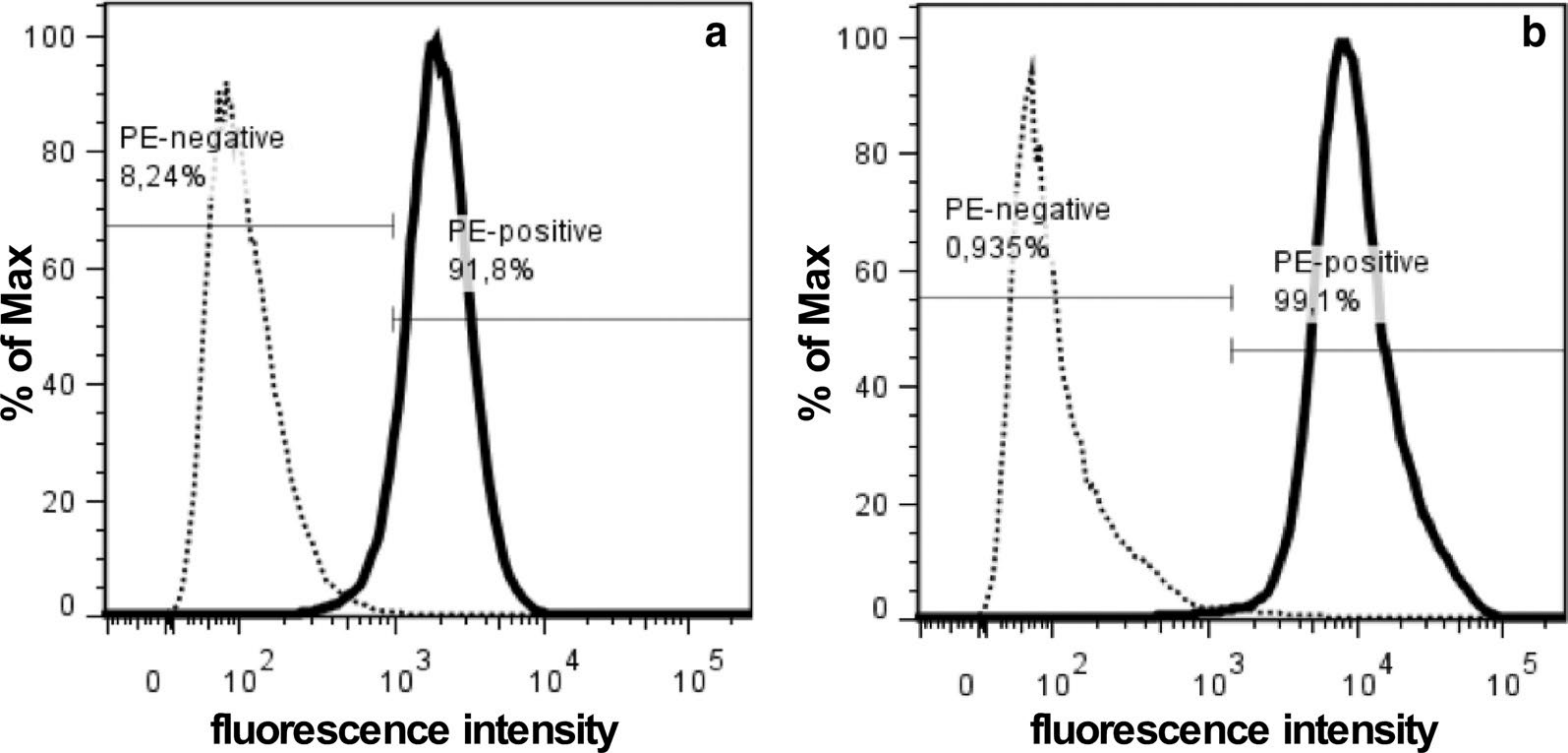
Cell surface expression of CD276. The density of CD276 on cell surfaces was enumerated by flow cytometry on normal urothelial cells (NUCs) and urothelial cancer cell lines (UCCLs). **a** The representative NUC (HL19/17) shows a mean fluorescence intensity (MFI) of 2E03 and 91.8% of cells were gated positive for CD276 (solid histogram). **b** The representative tumor cell line HT1197 shows a MFI of 1.12E04 and 99.1% of cells were gated CD276 positive. Dotted histograms present isotype controls. The x-axis denotes the fluorescence intensity, the Y-axis the number of cells recorded as % of the maximum

Transcript amounts encoding CD276 were enumerated in UCCLs and NUCs by quantitative RT-PCR. CD276 mRNA was detected in both populations but the mean expression in the 4 UCCLs was significantly higher than in the 4 NUCs (threefold, *p* < 0.006, Fig. 1a). Similarly, immunoblotting revealed a significantly higher expression of CD276 protein in UCCLs compared to NUCs (fivefold, *p* < 0.005, Fig. 1b). The mean fluorescence intensity (MFI) of cell surface CD276 expression as determined by flow cytometry was increased 3.7-fold in UCCLs (MFI: 13,302 ± 2163) when compared to NUCs (MFI: 3591 ± 2080; *p* < 0.012; Fig. 2).

### Expression of CD276 in bladder tumor tissue samples

**Fig. 3:**
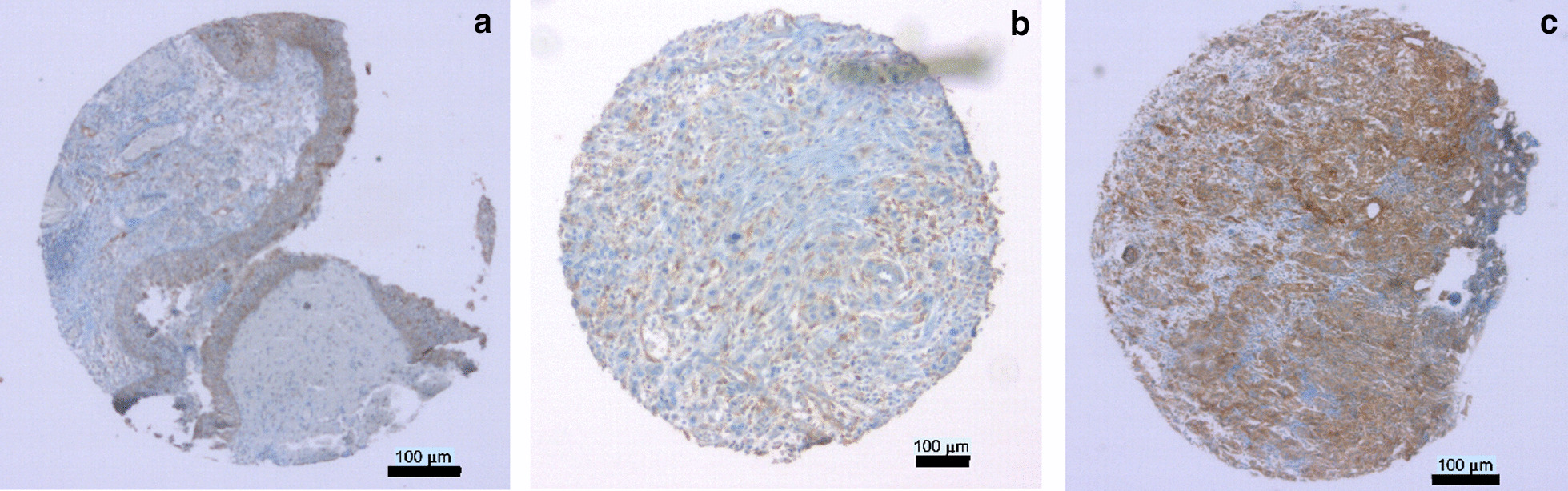
Detection of CD276 in tissue samples by immunohistochemistry. Representative tissue samples of benign urothelium (**a**), low expressing tumor tissue (**b**) and high expressing tumor tissue (**c**) were assessed for expression of CD276 on a tissue microarray by immunohistochemistry. Sections were counterstained with hematoxylin and eosin to visualize tissue structure. Size bars = 100 μm

**Fig. 4:**
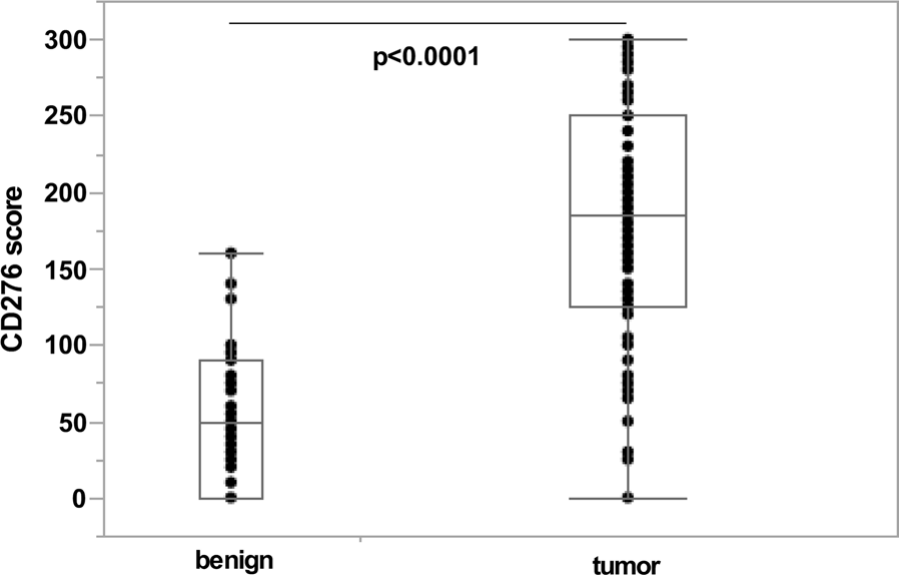
Expression of CD276 in normal appearing versus malignant bladder tissue. Paraffin sections of tissue samples from bladder cancer patients of all stages were reacted with anti-CD276 antibody and then counterstained by hematoxylin. The median CD276 expression score was significantly lower in areas surrounding the tumor and appearing healthy (score 50) when compared to the areas confirmed by an expert pathologist as tumor tissue (score 185, *p* < 0.0001). A considerable scatter of individual CD276 scores was noted in both cohorts

Tissue microarrays (TMA) were assembled from samples of 28 stage pT2, 46 stage pT3, 23 stage pT4 tumors and one additional BC tumor sample with unknown stage. The clinical and pathologic characteristics of the patient cohort are summarized in Table 3. Paraffin sections from tissue samples of these patients were stained with anti-CD276 antibody to determine the expression pattern of this immune checkpoint protein (Fig. 3). CD276 expression was found in urothelial cells but not in the connective tissue layers of histologically normal appearing bladder in bladder cancer patients (Fig. 3a). Low and dispersed CD276 expression was noted in some tumor samples (Fig. 3b), while its expression was high in other tumor samples (Fig. 3c). When comparing CD276 expression in healthy versus tumor tissue obtained from the 98 patient’s bladders, its median expression was significantly lower in normal appearing tissue surrounding the tumors (score 50, n = 47) when compared to the bladder tumors themselves (score 185, n = 98; *p* < 0.0001, Fig. 4). However, the CD276 expression scores were quite variable in both areas, in the tumors as well as the surrounding benigne tissue: the benign samples scored between 0 and 160, and tumor samples scored between 0 and 300.

**Table 3:** Patient characteristics

BC patients	98 total
Tumor samples—n (%)	98 (100.0)
Benigne samples—n (%)	47 (100.0)
Gender—n (%)	
Female	23 (23.5)
Male	75 (76.5)
Age, years	
Median (range)	69 (32–84)
pT-Stage—n (%)	
2a	15 (15.5)
2b	13 (13.6)
3a	22 (22.7)
3b	24 (24.7)
4a	17 (17.5)
4b	6 (6.2)
Unknown	1
Grading—n (%)	
1	0
2	23 (23.5)
3	75 (76.5)
Concomitant CIS—n (%)	
Yes	28 (30.1)
No	65 (69.9)
Unknown	5
cN-Stage—n (%)	
0	56 (59.5)
1	23 (24.5)
2	13 (13.7)
3	2 (2.3)
Unknown	4
cM-Stage—n (%)	
0	85 (90.4)
1	9 (9.6)
Unknown	4
R-Stage—n (%)	
0	80 (81.6)
1	15 (15.3)
2	3 (3.1)

However, the range of the CD276 score of benign samples surpassed the lowest quartile of the range of CD276 scores of the tumor samples (i.e., score 125) in only 3 of 47 cases (i.e., in 6.3%). In 12 of 47 benign samples (25%) CD276 expression was not observed at all (Fig. 4). Only 8 of 98 BC tumor samples (8.1%) scored within the range of the 3 lower quartiles of the benign samples (i.e., score < 90; Fig. 4).

**Fig. 5:**
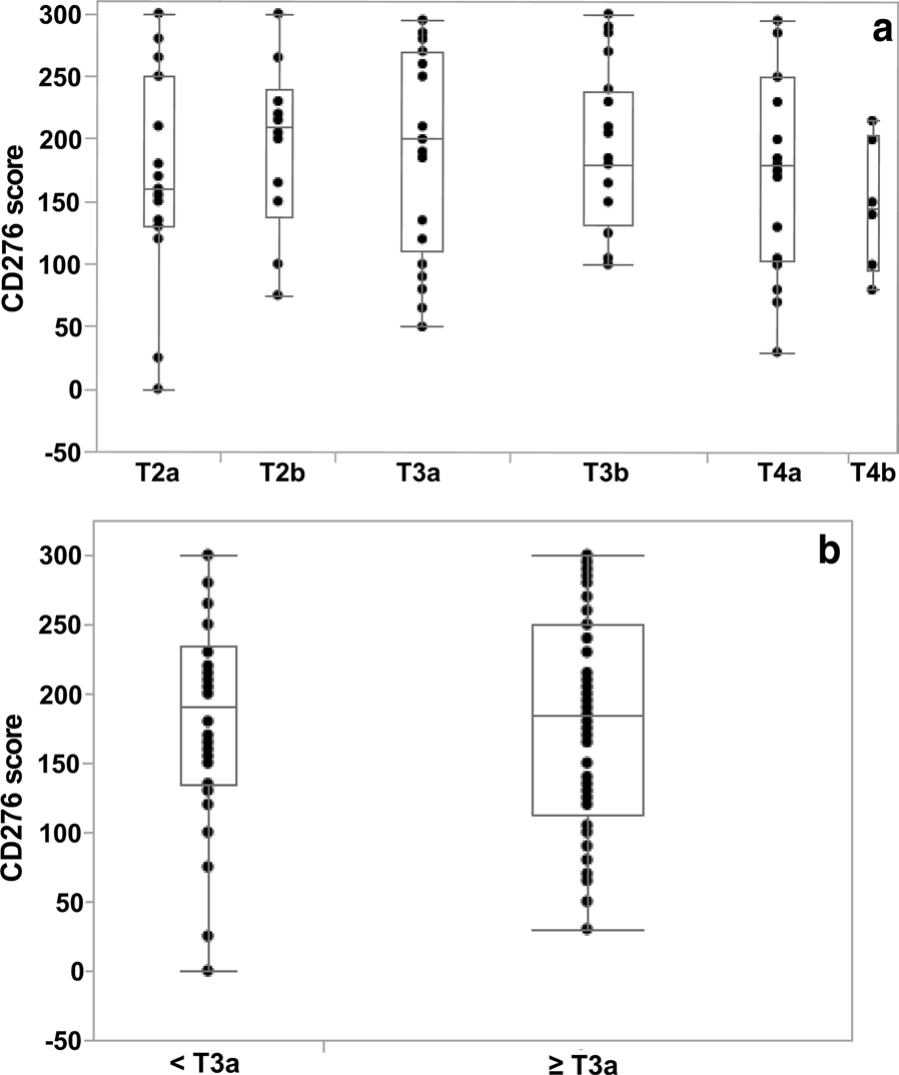
Expression of CD276 in bladder tumors in different tumor stages. **a** Bladder tumor samples from patients at stages T2a to T4b were investigated for CD276 expression and are presented as function of the clinical tumor stage. The median CD276 expression peaked in stage T2b samples (score 210), slightly declined throughout later stages to drop in tumor tissue samples obtained from late-stage patients at T4b to a median score of 145. **b** Bladder tumor samples of patients in all stages were investigated for CD276 expression and correlated to the tumor stages < pT3a versus ≥ pT3a. Significant differences in expression of CD276 in < pT3a (median score 190) versus ≥ pT3a tumors (median score 185; χ2 = 0.86) were not observed

**Fig. 6:**
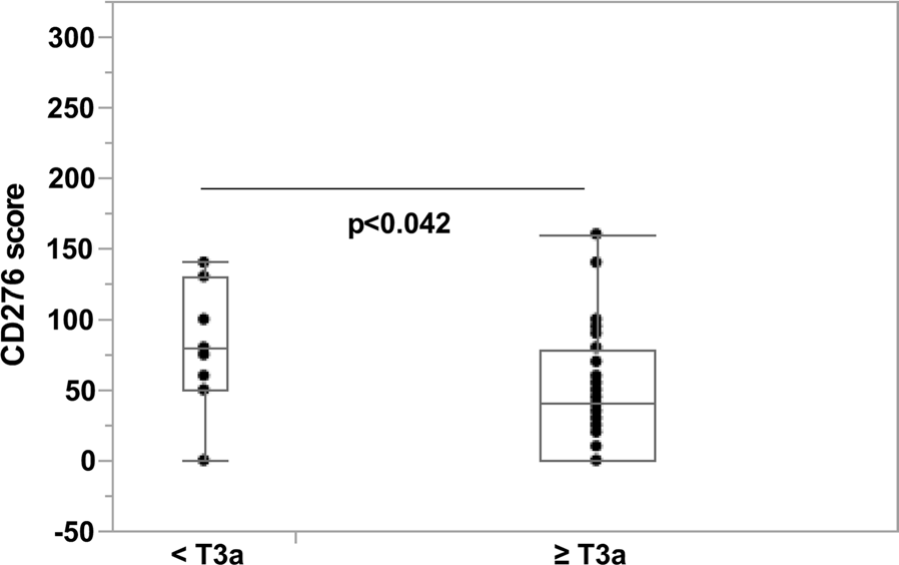
CD276 expression in normal appearing urothelium from bladder tumor patients. Expression of CD276 was evaluated by immunohistochemistry in areas presenting with normal histology and samples obtained from patients at earlier (< pT3a) versus later T stages (≥ pT3a) were compared. In confirmed normal urothelium from patients of tumor stages < pT3a, CD276 expression scored with a median of 80. In confirmed normal urothelium from tumor patients at stages ≥ pT3a CD276 dropped significantly to a median of 40

When correlating the CD276 expression to the tumor stage, elevated expression was found in all bladder tumor stages from T2a to T4b (Fig. 5a) when compared to the median expression score of normal appearing urothelial samples from the same patients (Fig. 4). The peak median expression intensity of 210 was measured in samples obtained from pT2b tumors (Fig. 5a). No significant differences were observed in CD276 expression when comparing organ-confined (< pT3a, median score 190) with locally advanced tumors (≥ pT3a, median score 185; χ2 = 0.86; Fig. 5B). However, a significant drop in the mean expression of CD276 was noted in the histologically normal tissue samples when stratified by the T stage of the tumor in the same specimen (< pT3a vs*.* ≥ pT3a, *p* < 0.042; Fig. 6). The median score of CD276 expression dropped from 80 in benign urothelium from < pT3a patients to 40 in stage ≥ pT3a samples (Fig. 6).

## Discussion

Elevated expression of CD276 in bladder cancer samples was reported previously [22] and success of experimental anti-CD276 antibody therapies raised hope for a new immune checkpoint therapy [6, 15, 16]. Our in vitro data using the well-established bladder cancer cell lines HT1197, TCCsup, RT4, and 5637 in comparison to four urothelial cell populations generated from explants from healthy ureters corroborated a significant overexpression of CD276 in BC cell lines. Significant overexpression of CD276 was also noted by immunohistochemistry in bladder tumors samples when compared to benign urothelium obtained from the same bladders. Slightly elevated CD276 scores in benigne samples from early-stage BC tumor patients could be caused by a moderate activation of CD276 expression in a few cells surrounding the tumor without showing the typical histology of a BC tumor by microscopy. CD276 transcript analyses of the corresponding malignant and benigne areas of BC samples could explore this hypothesis. Of note, healthy bladder tissue from healthy volunteers was not available. Therefore, mechanisms of CD276 regulation in normal appearing tissue surrounding tumors versus healthy tissue are beyond the focus of the current study.

When analyzing the association between CD276 expression and tumor stage by immunohistochemistry in sample subsets, the highest median scores were observed in T2b and T3a tumors, followed by a minor, non-significant decline in higher stages (T3b and T4). In contrast, in benign urothelium from samples of patients with later stage tumors (≥ T3a) mean CD276 was significantly reduced when compared to benign sites in sample from patients with early-stage tumors (< T3a).

Expression of CD276 in bladder tissue samples from cancerous and adjacent healthy areas was determined by immunohistochemistry but not, for instance, by transcriptomics or other molecular analyses. Therefore, the mechanisms contributing to the significant changes in CD276 expression in situ in the normal tissue remained obscure. Total protein expression is often regulated on the transcript level and tumor grade-depended elevation of CD276 transcripts and CD276 total protein were found in BC [22]. However, other mechanisms may contribute to the changes in CD276 expression observed as well. For instance, protease-mediated release of soluble CD276 was described on activated leukocytes [23]. Blocking the protease activity caused an accumulation of CD276 on these leukocytes [23]. Based on this finding we hypothesize that elevated expression of proteases by the bladder tumor cells may play a role in degradation of even little CD276 from the surface of the benign cells surrounding the actual tumor. This mechanism may act on bladder tumors as well, and thereby contribute to the minor reduction of the mean CD276 score observed in the pT4 samples. This hypothesis is in line with the finding that elevated expression of CD276 correlated with elevated expression of matrix metalloproteinases -2 and-9 [22].

We noted considerable variations of CD276 expression in individual samples from all stages of bladder tumors. This may be in part associated with changes in cell surface versus cytoplasmic expression of CD276. Such a conversion of CD276 expression has been observed in clear cell renal cell carcinoma [24]. Tumors with pronounced cytoplasmic expression were associated with worse prognosis when compared to those with membrane expression. But degradation of CD276 by proteases as mechanism to generate the cytoplasmic CD276 phenotype was not investigated in that study [24]. In other studies, tumor therapy by a PD-1 inhibitor reduced the expression of CD276 on bladder cancer carcinoma cells significantly in about 67% of samples investigated [25]. These findings indicated that CD276 expression may change its levels and patterns during the course of disease and/or depending on the therapy. However, the patients included in our study were not treated by immune checkpoint inhibitors.

A technical challenge is of course the fact that histology yields in many cases the sum of protein expression in the cytoplasm and on the cell surface. For the interaction with T lymphocytes as well as the direct binding of anti-CD276 antibodies or alike, the presentation of CD276 on the cells surface is critical. Therefore, additional studies need to determine the correlation between total protein expression in bladder tumor tissue in comparison to the CD276 density on tumor cell surfaces [22, 24, 25].

## Conclusions

Median expression of CD276 is significantly elevated in bladder carcinoma tissue but presents with a high inter-individual variability among patient samples. Diagnosis or therapy utilizing CD276 as target molecule should therefore be considered and evaluated with care, especially in late-stage patients.

## Data Availability

Data generated in this study are available from the corresponding author on reasonable request.

## Abbreviations

BC: Bladder cancer
FC: Flow cytometry
HRP: Horse reddish peroxidase
IB: Immunoblot, alias Western blot
IHC: Immunohistochemistry
KLH: Keyhole limped hemocyanin
mAB: Monoclonal antibody
MFI: Mean fluorescence intensity
NUCs: Normal urothelial cells
TMA: Tissue micro array
UCCL: Urothelial carcinoma cell line
